# When to Suspect Non-diabetic Kidney Disease in a Diabetic Patient?

**DOI:** 10.7759/cureus.28091

**Published:** 2022-08-17

**Authors:** Elenjickal Elias John, Sanjeet Roy, Jeethu Joseph Eapen, Rizwan Alam, Santosh Varughese

**Affiliations:** 1 Nephrology, Christian Medical College, Vellore, IND; 2 Pathology, Christian Medical College, Vellore, IND

**Keywords:** nodular glomerulosclerosis, kidney biopsy, clinical predictors, non-diabetic kidney disease, diabetes mellitus

## Abstract

The diagnosis of non-diabetic kidney disease (NDKD) in a diabetic patient has significant therapeutic and prognostic implications. There are certain proven clinical predictors of NDKD, which, when present in an appropriate clinical setting, would warrant a kidney biopsy. Herein, we describe four cases of NDKD diagnosed in rather unusual clinical settings, which add to the list of clinical predictors of NDKD. The first case was a “parainfectious glomerulonephritis” diagnosed in a 50-year-old diabetic woman who presented with persistent renal dysfunction despite successful treatment of urinary tract infection. The second case was “membranous nephropathy” diagnosed in a 43-year-old man with long-standing type 1 diabetes, which was associated with other microvascular complications. In this case, the only predictor was disproportionately low serum albumin. The third case was “amyloid light chain (AL) amyloidosis” diagnosed in an elderly diabetic who presented with progressive anasarca over six months. In this case, the only clinical predictor was a disassociation observed between urine dipstick and 24-hour protein estimation. In the fourth case, an elderly diabetic woman without underlying diabetic retinopathy presented with sudden onset nephrotic syndrome. A kidney biopsy was suggestive of diffuse nodular glomerulosclerosis. Immunofluorescence and electron microscopic evaluation were diagnostic of “gamma heavy chain deposition disease.” In all four cases, diagnosis of NDKD led to major therapeutic changes and attainment of renal remission. We have extensively reviewed all major biopsy cohorts of NDKD and have formulated an approach to the diagnosis of NDKD.

## Introduction

Diabetic kidney disease (DKD) occurs in 30-40% of diabetic patients of more than 10 years duration [1]. However, 45-80% of diabetics with kidney involvement undergoing kidney biopsy are diagnosed to have non-diabetic kidney disease (NDKD) [2-11]. There are certain proven clinical predictors of NDKD, which, when present in appropriate clinical settings, would warrant a kidney biopsy. Herein, we describe four unusual clinical situations wherein an NDKD was diagnosed, thus adding to the list of clinical predictors of NDKD.

## Case presentation

Case 1: superimposed infection-related glomerulonephritis (IRGN) on DKD

**Figure 1:**
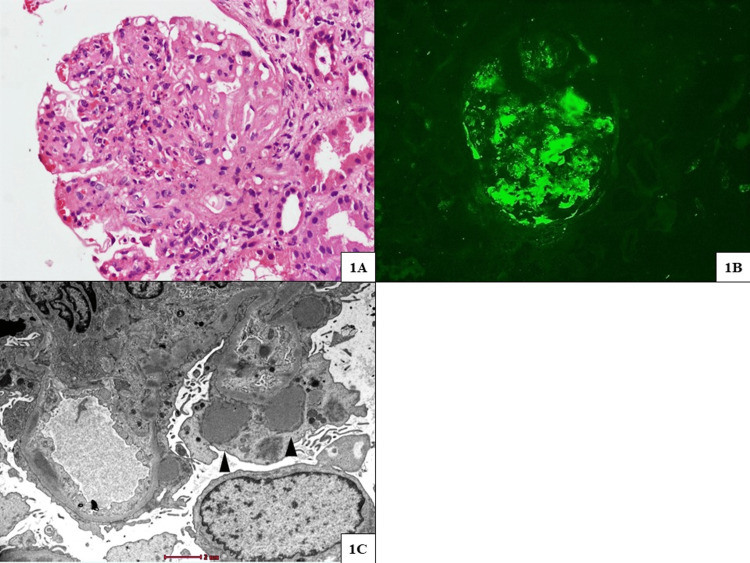
Renal biopsy findings of case 1 (infection-related glomerulonephritis) (A) Glomerulus with marked mesangial expansion, along with concomitant global endocapillary and exudative glomerulonephritis (hematoxylin and eosin stain, original magnification x200). (B) Immunofluorescence microscopy demonstrating granular mesangial and segmental capillary wall deposits of C3 in a "starry-sky pattern" (original magnification x400). (C) Mesangial, few small subendothelial, and predominantly large "hump-like" subepithelial electron-dense deposits (transmission electron microscope, original magnification x6000).

A 50-year-old woman with a long-standing history of type 2 diabetes mellitus (DM) presented with complaints of dysuria, oliguria, and anasarca of 10 days duration. On examination, her blood pressure (BP) was 160/90 mmHg, she had pitting pedal edema, and there was no renal angle tenderness. Her initial laboratory parameters were as follows: hemoglobin (Hb) of 10 g/dL, total leucocyte count of 15,600 cells/mm³, serum creatinine of 3.5 mg/dL, and serum albumin of 2.5 g/dL. Urine microscopy revealed protein at 1+, RBC at 15/HPF, WBC at 120/HPF, and 24-hour urine protein was 1.6 g/day. Urine culture grew *Escherichia coli *with >10^5^ CFU/ml. Non-contrast CT of the kidney, ureter, and bladder showed normal-sized kidneys with no features of pyelonephritis. She was treated with parenteral antibiotics and diuretics for 10 days. She improved symptomatically, though she needed two anti-hypertensives for BP control. Her repeat laboratory parameters after completion of the antibiotic course showed persistent renal dysfunction (serum creatinine of 5 mg/dL), microhematuria, sub-nephrotic proteinuria, hypocomplementemia (low C3 and normal C4), and sterile urine culture. She underwent a kidney biopsy with clinical suspicion of NDKD. Light microscopy (LM) showed features of diabetic nephropathy with superimposed endocapillary proliferation composed of neutrophil exudate. There was no neutrophil-rich interstitial infiltration suggestive of pyelonephritis. On immunofluorescence (IF), there were granular mesangial and capillary wall deposits of IgG (3+) and C3 (3+) in a “starry-sky pattern.” The ultrastructural evaluation showed the presence of electron-dense sub-epithelial humps (Figure 1). She was treated with a short course (six weeks) of oral prednisolone (1 mg/kg/day) with rapid tapering. After six weeks of treatment, her renal function improved (serum creatinine 1.5 mg/dL), complement levels normalized, and she was off anti-hypertensives.

Case 2: membranous nephropathy (MN)

**Figure 2:**
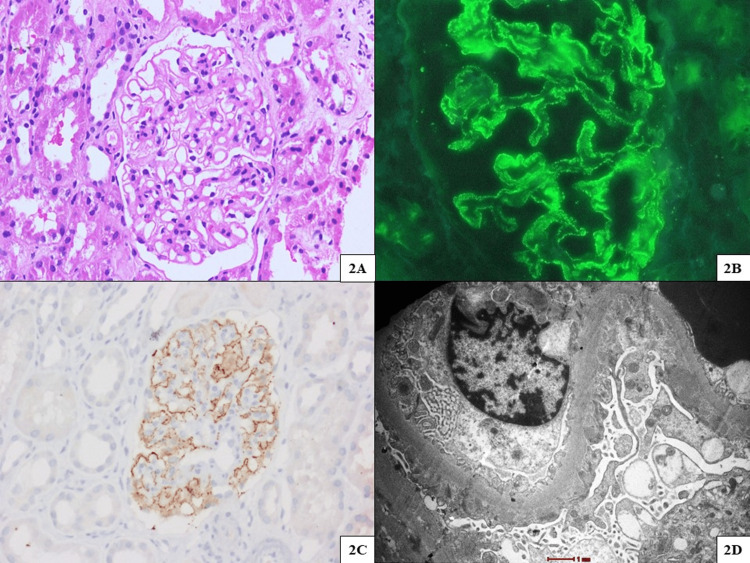
Renal biopsy findings of case 2 (membranous nephropathy) (A) Glomerulus with mild mesangial expansion, mild mesangial hypercellularity, and global uniform capillary wall thickening (hematoxylin and eosin stain, original magnification x400). (B) Immunofluorescence microscopy displaying fine granular global capillary wall staining for IgG (4+) (original magnification x400). (C) Glomerulus displaying phospholipase A2 receptor (PLA2R) immunohistochemistry positive staining (original magnification x400). (D) Capillary with numerous sub-epithelial to intramembranous electron-dense immune complex type deposits (transmission electron microscope, original magnification x6000).

A 43-year-old man with type 1 DM since the age of 20 years presented with anasarca, which was progressive over 12 months. His parameters were as follows: serum creatinine of 0.62 mg/dL, Hb of 14.4 g/dL, and albumin of 1.7 g/dL. Urine routine microscopy showed protein at 4+, RBC at 3-4/HPF, WBC at 3-4/HPF, 24-hour urine protein at 11.5 g/day, C3 at 116 mg/dL, and C4 at 24 mg/dL. Though he had an underlying proliferative diabetic retinopathy, he underwent a kidney biopsy in view of disproportionately low serum albumin. On LM, there was mesangial expansion and hypercellularity with global capillary wall thickening. On IF, there was diffuse granular capillary wall staining for IgG (4+), C3 (1+), kappa (3+), and lambda (1+). Electron microscopy (EM) showed electron-dense sub-epithelial deposits characteristic of MN (Figure 2). He was treated with rituximab infusions and attained partial remission over a period of six months.

Case 3: amyloid light chain (AL) amyloidosis

**Figure 3:**
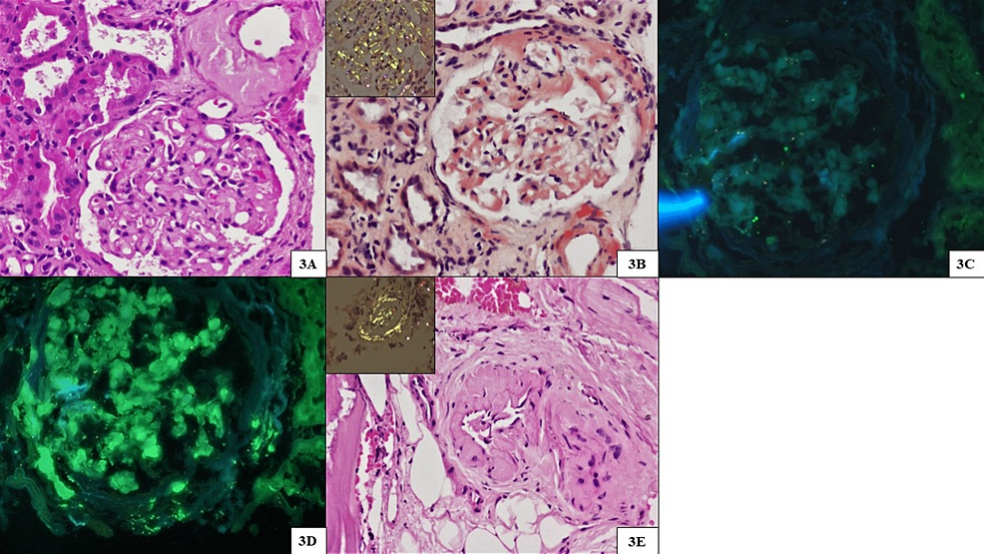
Renal and bone marrow biopsy findings of case 3 (amyloid light chain amyloidosis) (A) Glomerulus with mesangial expansion and deposition of large, periodic acid-Schiff (PAS)-negative eosinophilic amorphous deposits. Adjacent arteriole also shows similar transmural deposits (PAS stain, original magnification x200). (B) Congo red stain highlights mesangial congophilic deposits with apple green birefringence on polarizing microscopy (inset) (original magnification x200). Immunofluorescence microscopy highlighting kappa negativity (C) and abundant smudgy mesangial lambda light chain staining (D) (original magnification x400). (E) Bone marrow trephine biopsy highlighting arterioles with similar transmural pale eosinophilic deposits displaying apple green birefringence on polarizing microscopy (inset) (hematoxylin and eosin stain, original magnification x400).

A 69-year-old man with a history of type 2 DM for 10 years presented with anasarca, which was progressive over six months. On examination, he had a BP of 100/70 mmHg and pitting edema on both legs. His laboratory values were as follows: serum creatinine of 1.74 mg/dL, Hb of 15.2 g/dL, and albumin of 2.7 g/dL. Urine routine microscopy showed albumin trace, RBC at 3-4/HPF, WBC at 1-2/HPF, 24-hour urine protein at 4.8 g/day, and serum protein electrophoresis showed no M spike. He underwent a kidney biopsy due to disassociation observed between urine dipstick and 24-hour urine protein estimation. There was marked mesangial expansion by pale amorphous eosinophilic deposits on LM. These deposits were periodic acid-Schiff (PAS) pale, silver negative, and congophilic with apple green birefringence on polarized microscopy. IF showed lambda restricted smudgy staining (3+) within the glomerular mesangium, arterial wall, and focal interstitium. Bone marrow biopsy showed an arteriole with scanty intramural congophilic deposits with apple green birefringence (Figure 3). Serum immunofixation electrophoresis showed IgG lambda monoclonality. He was initiated on a “CyBorD” (cyclophosphamide, bortezomib, and dexamethasone) induction regimen and attained a very good partial response (VGPR) at six months.

Case 4: gamma heavy chain deposition disease (HCDD)

**Figure 4:**
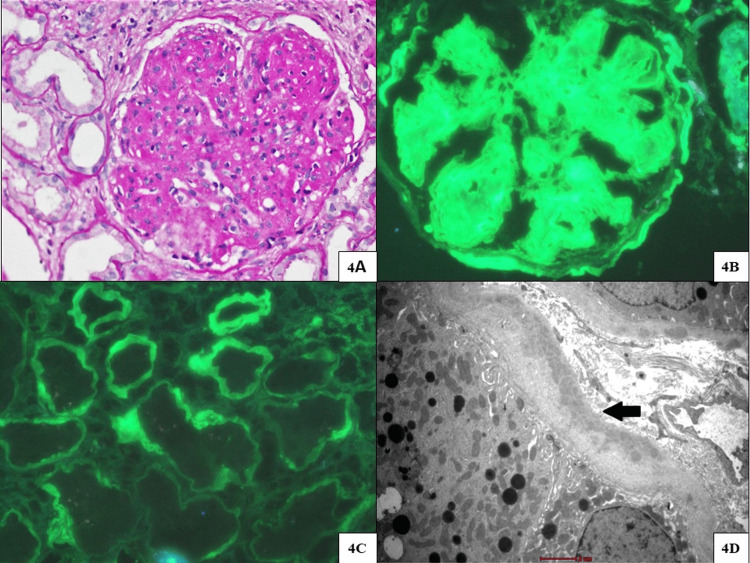
Renal biopsy findings of case 4 (gamma heavy chain deposition disease) (A) Glomerulus with marked mesangial expansion with nodular condensation with periodic acid-Schiff (PAS)-positive staining, mild mesangial hypercellularity, and segments of mild capillary wall thickening (PAS stain, original magnification x200). (B and C) Immunofluorescence microscopy for IgG highlighting strong (4+ intensity) staining in the glomerular mesangium, global, capillary walls, Bowman’s capsule, and linear staining of tubular basement membranes (original magnification x400). (D) Tubules displaying fine to coarse powdery basement membrane deposits (transmission electron microscope, original magnification x6000).

A 61-year-old woman with a history of diabetes for four years presented with sudden onset anasarca. Her initial laboratory parameters were as follows: Hb of 8.7 g/dL, serum creatinine of 2.8 mg/dL, albumin of 2.7 g/dL, and normal serum protein electrophoresis. Urine routine microscopy showed protein at 3+, RBC at 2-3/HPF, WBC at 2-3/HPF, and 24-hour urine protein was 14.3 g/day. She underwent a kidney biopsy due to the absence of retinopathy, well-controlled glycemic status, and a sudden onset of nephrotic syndrome. On LM, there was a mesangial expansion with diffuse nodular glomerulosclerosis involving all the glomeruli. These nodules were PAS positive, silver negative, and Congo negative. IF showed strong linear staining for IgG (4+) along the tubular basement membrane and blood vessels along with smudgy mesangial staining for IgG (4+), C3 (2+), and C1q (3+). There was only trace capillary wall staining for kappa and lambda. On EM, fine powdery deposits were also seen along the lamina rara interna of the glomerular basement membrane and within the mesangium (Figure 4). Bone marrow biopsy showed mild CD138-positive plasmacytosis (15-20%) with no light chain restriction seen on immunohistochemistry. She was initiated on a “CyBorD” induction regimen and attained partial remission of proteinuria within three months of therapy.

## Discussion

DKD occurs in 30-40% of patients with more than 10 years duration of DM. Clinically, these patients progress slowly over years from an initial stage of hyperfiltration (increase in estimated glomerular filtration rate (eGFR)) to microalbuminuria (now termed as moderately increased proteinuria) to macroalbuminuria (now termed as severely increased proteinuria) to overt nephropathy and finally progress to kidney failure. The histological features of DKD include thickening of the glomerular capillary wall, mesangial expansion, nodular glomerulosclerosis (Kimmelstiel-Wilson lesions), diffuse glomerulosclerosis, hyaline arteriosclerosis, and exudative lesions like fibrin caps, capsular drops, and hyaline thrombi [1]. Most of these lesions are irreversible and current therapies include only strategies to slow down the progression by tight control of blood sugars and blood pressure. Antiproteinuric measures like renin-angiotensin-aldosterone blockers and sodium-glucose cotransporter inhibitors have proven efficacy in reducing the rate of disease progression [12].

The most commonly diagnosed NDKD etiologies include primary glomerular diseases (MN and IgA nephropathy), secondary glomerular diseases (IRGN and antineutrophil cytoplasmic antibody-associated vasculitis), and tubulointerstitial diseases (acute interstitial nephritis and acute tubular necrosis) (Table 1).

**Table 1:** Comparison between major cohorts of non-diabetic kidney disease AAV, antineutrophil cytoplasmic antibody-associated vasculitis; eGFR, estimated glomerular filtration rate; DKD, diabetic kidney disease; DM, diabetes mellitus; DPGN, diffuse proliferative glomerulonephritis; GBM, glomerular basement membrane; GN, glomerulonephritis; HbA1c, glycated hemoglobin; HSP, Henoch-Schonlein purpura; IRGN, infection-related glomerulonephritis; MGRS, monoclonal gammopathy of renal significance; MPGN, membranoproliferative glomerulonephritis; NDKD, non-diabetic kidney disease; SD, standard deviation; TBMN, thin basement membrane nephropathy; TMA, thrombotic microangiopathy.

Author	John et al. [2]	Das et al. [3]	Bermejo et al. [4]	Bi et al. [5]	Soni et al. [6]	Liu et al. [7]	Bermejo et al. [8]	Liu et al. [9]	Sharma et al. [10]	Fontana et al. [11]
Cohort duration	1985-1993	1990-2008	1990-2013	1999-2009	2000-2004	2000-2015	2002-2014	2004-2014	2011	2010-2020
Country	Vellore, India	Hyderabad, India	Spain	China	Hyderabad, India	China	Multicenter, Spain	Multicenter, China	USA	Italy
Total number of diabetic patients	80	75	110	220	160	273	832	1604	620	142
Age (years)	47.4 ± 10.2	45 ± 10.2	62 (50-74)	51.3 (30-79)	51.3 (30-79)	51.1 ± 12.4	61.7 ± 12.8	51.4 ± 11.4	62 (52-69)	62.7 ± 12.3
Male to female ratio	1.5:1	3.1:1	3.8:1	2.3:1	2.1:1	1.7:1	2.9:1	1.3:1	1.5:1	2.6:1
Type of diabetes mellitus (%)										
Type 1	0	0	3	0	0	0	7	4	2	4
Type 2	100	100	97	100	100	100	93	96	98	96
Duration of DM (years)	-	1 (1-15)	11 (1-20)	-	-	4.8 ± 5.7	10.8 ± 8.6	-	10 (5-15)	12 ± 10
Microvascular complications										
Diabetic retinopathy	17	0	22	65	62	14	27	-	80	34
Peripheral neuropathy	-	-	-	-	-	-	-	-	-	-
Hypertension (%)	-	84	-	-	-	42	87	-	-	-
HbA1c (%, mean ± SD)	-	-	-	-	-	-	-	-	-	4.7 ±1.3
Dyslipidemia (%)	-	-	-	-	-	69	-	-	-	58
Kidney function at biopsy										
Serum creatinine (mg/d)	-	3.1 (0.3-12)	2.6 (0.9-4.3)	-	-	-	2.8 ± 2.2	-	2.5 (1.6-4.4)	-
eGFR (ml/min/1.73m²)	-	-	-	-	-	-	38 ± 27	-	29 (14-54)	36 ± 27
Urine examination										
Microhematuria (%)	-	32	37	40	6	69	35	0.7	-	63
24-hour urine protein (g/day)	-	3.1 (0.3-12)	3.5 (0.5-6.5)	-	-	4.8 ± 4.2	2.7 (1.2-5.4)	-	4.3 (1.9-8)	3.9 (1.9-6.9)
Nephrotic range proteinuria (%)	42	39	-	-	34	28	-	51	-	41
Serum albumin (g/dL, mean ± SD)	-	-	-	-	-	-	-	-	-	3.3 ± 0.8
Renal histology (%)										
Diabetic kidney disease	19	36	34	55	28	25	39	45	37	37
Non-diabetic kidney disease	60	60	62	0	42	64	50	49	36	43
Superimposed NDKD on DKD	21	4	4	45	30	11	11	6	27	20
Cause of NDKD (%)										
Primary glomerular diseases										
Minimal change disease	18	13	3	4	5	3	2	10	0	1
Focal segmental glomerulosclerosis	11	6	7	4	8	1	3	5	18	1
Membranous nephropathy	9	10	6	22	11	28	5	40	6	19
IRGN/DPGN	21	19	0	8	17	4	0	2	1	4
C3 glomerulonephritis	0	0	0	0	0	0	0	0	0	2
Mesangioproliferative GN	6	0	3	14	4	7	2	0	0	6
IgA nephropathy/HSP	9	7	13	34	3	20	5	22	9	7
MPGN	0	2	3	0	1	0	3	1	0	7
Secondary glomerular diseases										
Lupus nephritis	1	6	1	2	1	4	1	1	0	0
AAV/anti-GBM/crescentic GN	8	10	5	2	5	5	3	1	5	3
Amyloidosis	3	0	3	0	1	2	2	0.5	3	0
Tubulointerstitial diseases										
Tubulointerstitial nephritis	8	2	7	0	22	5	6	3	5	4
Acute tubular injury	5	4	0	0	1	1	3	2	28	3
Renovascular disease	0	0	0	0	0	0	0	0	0	9
Others										
Hypertensive nephrosclerosis	0	0	0	4	6	12	9	3	18	0
MGRS	0	4	4	0	2	0	2	0	3	7
TMA	0	0	0	0	2	3	1	0	0	2
Atheroembolic kidney disease	0	0	1	0	3	0	1	0	1	0
Lipoprotein glomerulopathy	0	0	0	0	0	1	0	0	0	0
Pyelonephritis	0	0	0	0	3	0	0	0	0	0
Papillary necrosis	0	0	0	0	2	0	0	0	0	0
TBMN	0	0	0	0	0	0	0	0.5	0	0
Clinical predictors of NDKD										
Absence of diabetic retinopathy	Yes	No	Yes	Yes	Yes	Yes	Yes	No	No	Yes
Absence of hypertension	No	Yes	No	No	No	Yes	No	No	No	No
Shorter duration of diabetes mellitus	No	No	Yes	No	Yes	Yes	No	No	Yes	No
Microhematuria	No	No	No	Yes	No	No	Yes	Yes	No	No
Older age at presentation	No	No	Yes	No	No	No	Yes	Yes	No	No
Lower eGFR level	No	No	Yes	No	No	No	No	No	No	No
Higher eGFR level	No	No	No	Yes	No	No	No	No	No	No
Lower degree of proteinuria	No	No	Yes	No	Yes	Yes	No	No	No	No
Higher degree of proteinuria	No	No	No	Yes	No	No	No	No	No	No
Low serum complements	No	No	No	No	No	No	No	No	Yes	No
Presence of monoclonal gammopathy	No	No	No	No	No	No	No	No	Yes	Yes
Significant change in therapy after kidney biopsy (%)	-	-	-	-		-	-	-	30	32
Kidney failure (%)										
DKD	-	-	11	-	-	-	50	-	-	49
NDKD	-	-	22	-	-	-	28	-	-	16
Superimposed NDKD on DKD	-	-	-	-	-	-	45	-	-	32
Death (%)										
DKD	-	-	-	-	-	-	25	-	-	-
NDKD	-	-	-	-	-	-	18	-	-	-
Superimposed NDKD on DKD	-	-	-	-		-	26	-	-	-

Diagnosis of NDKD has got both therapeutic as well as prognostic implications. It leads to a significant change in therapy in almost one-third of biopsied patients [10,11]. Kidney failure and mortality rates are lower in diabetic patients with an underlying NDKD [8]. In a prospective, multicenter observational cohort study involving 2,484 diabetic patients conducted in Japan, 281 (11.3%) progressed to end-stage kidney disease (ESKD) over a period of 4.4 years. The hazard ratios for the development of ESKD were 7.1 (2.5-20.5, 95% CI) and 0.9 (0.2-4.2, 95% CI) for DKD and NDKD patients, respectively. The annual decline in eGFR in DKD patients (-9.7 ml/min/1.73 m²) was larger than in NDKD patients (-4 ml/min/1.73 m²) [13]. In a biopsy cohort of 119 diabetic patients, 36%, 54%, and 19% of patients had DKD, NDKD, and superimposed NDKD on DKD, respectively. During the follow-up period, 33 (28%) developed ESKD. The predictors of ESKD on multivariate analysis were the presence of DKD, longer duration of DM, high baseline creatinine levels, and high systolic BP [14].

Kidney biopsy is the most crucial tool to diagnose NDKD. However, with an unrestricted biopsy policy, 50% will have pure DKD, whereas, with a restricted biopsy policy based on clinical indications, two-thirds of patients will have pure NDKD [15]. This signifies the relevance of knowing the clinical predictors of NDKD and how to apply them in an appropriate clinical setting (Table 2).

**Table 2:** Clinical predictors of non-diabetic kidney disease ANA, antinuclear antibody; ANCA, antineutrophil cytoplasmic antibody; DM, diabetes mellitus; eGFR, estimated glomerular filtration rate; MIDD, monoclonal immunoglobulin deposition disease; NRP, nephrotic range proteinuria; PAS, periodic acid-Schiff; PLA2R, phospholipase A2 receptor.

Clinical predictors of non-diabetic kidney disease
(1) Absence of microvascular complications of diabetes [2,4-8,11]
Proteinuria (>1 g) or renal dysfunction without diabetic retinopathy in patients with type 1 DM
(2) Short duration of diabetes [4,6,7,10]
Proteinuria (>1 g) or renal dysfunction, which occurs within five years of onset of diabetes, especially in patients with type 1 DM
(3) Absence of hypertension [3,7]
Proteinuria (>1 g) or renal dysfunction without hypertension
(4) Nephrotic range proteinuria [5]
Sudden onset of NRP
NRP with marked hypoalbuminemia
NRP with elevated serum anti PLA2R antibody titers
(5) Renal dysfunction [4]
Decline in eGFR by >10ml/min/1.73 m²/year
Renal dysfunction without proteinuria (suspicion of renal artery stenosis)
(6) Glycemic control [2]
Progression of proteinuria or rapid decline in eGFR despite aggressive glycemic control
(7) Nephritic syndrome [5,8-10]
Glomerular hematuria, acanthocytes, and/or red blood cell casts
Low complement levels
Positive serological markers like ANA or ANCA
Persistent renal dysfunction despite eradication of infection source
(8) Suspicion of monoclonal gammopathy [10,11]
Discrepancy between urine dipstick and 24-hour urine protein estimation
Detectable circulating monoclonal immunoglobulin by serum protein electrophoresis, immunofixation electrophoresis, or serum free light chain assay
(9) Atypical nodular glomerulosclerosis on kidney biopsy [1]
Nodules are uniformly distributed within all glomeruli
PAS positive, silver negative, and Congo red negative (MIDD)
PAS positive, silver negative, and Congo red positive (amyloidosis)

The most significant predictor of NDKD is the absence of diabetic retinopathy [2,5-8]. This retinal-kidney bond in diabetes is due to common underlying risk factors for the development of both nephropathy and retinopathy [16]. The positive predictive value for NDKD in type 2 DM patients with and without retinopathy is 54% and 87%, respectively [2]. The combination of absent retinopathy with nephrotic range proteinuria (NRP) or microhematuria is a stronger predictor of NDKD [2,6]. Another important predictor of NDKD is the short duration (< five years) of diabetes. NDKD is highly unlikely with a duration of diabetes >12 years [10]. Though some studies have shown microhematuria as a predictor of NDKD [5,8,9], 33-50% of patients with DKD may also have microhematuria [17]. Sudden onset NRP (without going through stages of micro and macroalbuminuria) should raise the suspicion of underlying podocytopathy. Renal dysfunction without significant proteinuria in a diabetic patient should raise suspicion of renal artery stenosis. The eGFR declines by approximately 1 ml/min/1.73 m²/year after the third decade of life. An annual decline of eGFR by >10 ml/min/1.73 m² in a diabetic patient should raise suspicion of NDKD.

IRGN is the most common cause of NDKD in the tropical world [2,3]. Parainfectious glomerulonephritis is a subtype of IRGN commonly seen in diabetics, which occurs with an ongoing infection (lung or urinary tract) caused by gram-negative organisms [18]. It should be suspected in diabetics who have persistent renal dysfunction despite eradication of infection source as in our first case. Hypoalbuminemia is unlikely in secondary glomerular diseases such as DKD or secondary focal segmental glomerulosclerosis. This may be due to the very slow appearance of proteinuria, which allows compensatory mechanisms to counterbalance the protein losses [19]. This was the only clue to the diagnosis of NDKD in our second case. Future studies are, however, needed to evaluate hypoalbuminemia as a predictor of NDKD in the setting of NRP. Urine dipstick for proteinuria specifically detects albumin. A disassociation between urine dipstick (absent or trace) and 24-hour protein estimation (NRP) should raise the suspicion of tubular or overflow proteinuria (Bence Jones protein). A simple but often overlooked observation such as this led to the diagnosis of AL amyloidosis in our third case.

Nodular glomerulosclerosis is not unique to DKD as it is seen in a variety of other conditions like amyloidosis, monoclonal immunoglobulin deposition disease (MIDD), fibrillary glomerulopathy, immunotactoid glomerulopathy, chronic hypoxic conditions (cyanotic heart disease, cystic fibrosis, and Takayasu arteritis), and idiopathic nodular glomerulosclerosis in smokers. Nodular glomerulosclerosis in DKD (known as Kimmelstiel-Wilson lesions) is irregularly and peripherally distributed, eosinophilic on hematoxylin and eosin (H&E) stain, PAS positive, argyrophilic (silver positive), and is associated with exudative lesions, glomerular basement membrane thickening, and afferent/efferent arteriolar hyalinosis. Whereas, nodular glomerulosclerosis in MIDD is regularly distributed within glomeruli, eosinophilic on H&E stain, PAS positive, and silver negative [1]. Etiological confirmation as in our fourth case of HCDD requires EM, which showed characteristic electron-dense granular, powdery deposits along the glomerular and tubular basement membrane and within mesangial nodules. EM is thus a crucial investigation, which should be thus included in the diagnostic armamentarium of NDKD. In all four cases, diagnosis of NDKD led to major therapeutic changes and attainment of renal remission.

## Conclusions

The traditional clinical predictors of NDKD include the absence of diabetic retinopathy, short duration of diabetes, sudden onset NRP, rapid decline in eGFR, nephritic syndrome, and hypocomplementemia. We would like to add persistent renal dysfunction despite eradication of infection source, NRP with severe hypoalbuminemia, disassociation between dipstick and 24-hour protein estimation, and atypical nodular glomerulosclerosis to this list. The diagnosis of NDKD in a diabetic patient has significant therapeutic and prognostic implications.
